# Flavonoid‐enriched extract from *Millettia speciosa* Champ prevents obesity by regulating thermogenesis and lipid metabolism in high‐fat diet–induced obese C57BL/6 mice

**DOI:** 10.1002/fsn3.2664

**Published:** 2021-11-27

**Authors:** Mao‐Yuan Wang, Wen‐Yu Ma, Qing‐Long Wang, Qing Yang, Xiao‐Xia Yan, Huan Tang, Zhi‐Ying Li, Ying‐Ying Li, Shi‐Xiu Feng, Zhu‐Nian Wang

**Affiliations:** ^1^ Chinese Academy of Tropical Agricultural Sciences/Key Laboratory of Crop Gene Resources and Germplasm Enhancement in Southern China Tropical Crops Genetic Resources Institute Ministry of Agriculture Haikou China; ^2^ Tropical Wild Plant Gene Resource Ministry of Agriculture/National Genebank of Tropical Crops Danzhou China; ^3^ Key Laboratory of South Subtropical Plant Diversity Fairy Lake Botanical Garden Shenzhen & Chinese Academy of Sciences Shenzhen China

**Keywords:** browning of white adipocyte, flavonoids, lipid metabolism, *Millettia speciosa* Champ, obesity, thermogenesis

## Abstract

*Millettia speciosa* (*M. speciosa*) Champ is a medicinal and edible plant. The roots are rich in flavonoids, which possess multiple biological activities, including lipid‐lowering effects. This study aimed to explore the effect of flavonoid‐enriched extract from *M. speciosa* (FMS) on obesity. The UPLC‐Q‐TOF‐MS analysis and chromatographic analysis were adopted to identify flavonoid compounds in FMS. Male C57BL/6J mice were fed with a high‐fat diet for 3 months and were then treated with FMS (50 or 100 mg/kg/d) or Orlistat (10 mg kg^−1^ d^−1^) for another 8 weeks. A total of 35 flavonoids were identified in the extract of *M. speciosa* root. FMS reduced body weight gain, liver weight gain, white adipose tissue, lipid accumulation, and blood glucose. The levels of TG, ALT, AST, and inflammatory‐related adipokines (TNF‐α and IL‐6) in serum were also reduced by FMS. In addition, FMS promoted thermogenesis in brown adipose tissue and induced the activation of lipolysis, fatty acid oxidation, and oxidative phosphorylation in white adipose tissues. In summary, long‐term administration of FMS could ameliorate high‐fat diet–induced obesity by stimulating adipose thermogenesis and lipid metabolism.

## Graphical abstract

*Millettia speciosa* Champ is a traditional Chinese medicine with its root as an edible food. For the first time, our study suggests that long‐term administration of FMS ameliorates obesity by stimulating adipose thermogenesis and lipid metabolism.

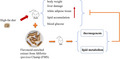

## 1 INTRODUCTION

Obesity is characterized by an excess of adipose tissue and apparent overweight (Vargas‐Castillo et al., 2017). It has been reported that in 2016, more than 1.9 billion adults (older than 18 years) were overweight; of these, over 650 million were obese (Tsatsoulis & Paschou, 2020). In addition, about one million children were overweight or obese, who were under the age of 5 (data from the World Health Organization) (Tsatsoulis & Paschou, 2020). Obesity is regarded as a chronic metabolic disease condition associated with serious health risks, such as diabetes, hypertension, insulin resistance, and cardiovascular disease (Acosta et al., 2016; Gyllenhammer et al., 2016; Lavie et al., 2016). It badly affects human quality of life and health, and reduces life expectancy (Seidell & Halberstadt, 2015). Therefore, the prevention of obesity is a great challenge of society today.

Mammals have two specialized types of adipose tissue, that is, white adipose tissue (WAT) and brown adipose tissue (BAT), which serve opposite functions. WAT accounts for 25% of the body weight of women, whereas it is 20% in men. It is an active endocrine organ that is responsible for the release of fatty acids and stores energy in the form of fat (Trayhurn & Beattie, 2001). BAT is histologically distinct from WAT, as it is composed of multilocular lipid droplets and huge amounts of mitochondria (Holstila et al., 2017). BAT consumes energy by oxidizing fatty acids to generate heat (Qurania et al., 2018). Importantly, browning of WAT promotes energy expenditure through triggering thermogenesis, which suppresses high‐fat diet (HFD)–induced weight gain (Bi et al., 2014; Cong et al., 2018). The current research suggests that browning of WAT might serve as a novel strategy for the prevention of obesity (Baskaran et al., 2016; Guan et al., 2018).

*Millettia speciosa* (*M. speciosa*) Champ belongs to the Leguminosae family (Zhao et al., 2015). It is widely distributed in the Guangdong, Guangxi, and Hainan Provinces of China. Its roots are utilized as a folk medicine by locals to treat rheumatic arthritis and irregular menstruation (Chen et al., 2015; Zhao et al., 2017). An experimental study has shown its effects in inhibiting tuberculosis, chronic hepatitis, leukorrhagia, and other diseases (Fu et al., 2016). In addition, its roots are commonly used as an ingredient in soup in the south of China. The polysaccharide fraction of *M. speciosa* root has been reported to have immunomodulatory activity as functional food (Huang et al., 2020). It has recently been listed as a herb for both edible and medicinal applications. Previous investigations into chemical constituents of *M. speciosa* state more than 50 compounds were isolated, which were identified as flavonoids, alkaloids, terpenoids, phenylpropanoids, and sterols (Yu, & Liang, 2018; Fu et al., 2016). Moreover, increasing evidence has indicated that flavonoids are the main constituents contributing to the bioactivities of *M*. *speciosa* extract (Zhao et al., 2015, 2017). Flavonoids have been reported to possess anti‐obesity effects and lipid‐lowering effects (Nery et al., 2021; Song et al., 2019). Formononetin is an isoflavone; as the signature ingredient of *M. speciosa* root, it could inhibit adipogenesis and HFD‐induced obesity through increasing adipocyte thermogenesis (Gautam et al., 2017; Nie et al., 2018). What’s more, a flavonoid‐enriched extract from *Hippophae rhamnoides* seed exhibited anti‐hypertensive and anti‐obesity effects (Yang et al., 2017). Flavonoid‐enriched orange peel extract showed anti‐diabetic activity and anti‐inflammatory effects (Gosslau et al., 2018). However, the effect of flavonoid‐enriched extract from *M*. *speciosa* (FMS) on obesity has not been assessed earlier. Hence, in our study, the effect of FMS on weight, glucose tolerance, and insulin sensitivity was investigated, and its possible role in lipid synthesis in the liver and WAT of HFD‐induced C57BL/6 obese mice was assessed. In addition, its effects on WAT browning, thermogenic programming, and lipid metabolism in obese mice were also explored.

## 2 MATERIALS AND METHODS

### 2.1 Plant materials

The roots of *M. speciosa* were collected from the germplasm repository of tropical medicinal plants, Danzhou City, Ministry of Agriculture, Hainan Province, China, in November 2019. The voucher specimens were authenticated by Zhunian Wang from Tropical Crops Genetic Resources Institute, Chinese Academy of Tropical Agricultural Sciences, and were deposited in Key Laboratory of Crop Gene Resources and Germplasm Enhancement in southern China, Ministry of Agriculture, China, Hainan, Haikou (No. B20191121).

### 2.2 Preparation of flavonoid‐enriched extract from *M. speciosa*

Supercritical fluid extraction was conducted using an HA120‐50‐01SFE system (Hua'an SFE, Inc.) to determine the total flavonoid fraction from the roots of *M*. *speciosa*. One kilogram of the roots of *M*. *speciosa* was powdered and filtered through a 40‐mesh sieve. For each extraction, 500 g of the powder was placed into the cylindrical extractor vessel with 95% ethanol as the co‐solvent. The extraction temperature was set at 45°C after the loaded extractor vessel was assembled. By the time the temperature reached at 45°C, pressurized gas from a CO_2_ cylinder was introduced into the compressor. When the pressure reached 25 MPa, the extraction was timed and lasted for 20 min. The procedure was conducted 20 times, and the yielded extract (FMS, 52.0 g) was collected and combined for isolation, purification, and the UPLC‐Q‐TOF‐MS analysis of flavonoids.

### 2.3 UPLC‐Q‐TOF‐MS analysis

Chromatographic conditions: Separation the UPLC analysis was conducted on a Waters ACQUITY UPLC system (Waters, Milford, MA, USA) with an ACQUITY UPLC HSS T3 C18 column (2.1 × 100 mm 1.8 μm). A solvent mixture of 0.1% aqueous formic acid (A) and acetonitrile with 0.01% formic acid (B) was applied. The flow rate was 0.3 ml/min, and each sample solution was injected with 1 μl. The column temperature was controlled at 40°C. A linear gradient elution was used as follows: 0–1 min, 6%–18% B; 1–2 min, 18%–20% B; 2–6 min, 20%–22% B; 6–9 min, 22%–30% B; 9–12 min, 30%–36% B; 12–16 min, 36%–99% B; 16–19 min, 99%–99% B, 19–20 min, 99%–6% B, 20–23 min, 6%–6% B. Three injections were performed for each sample and blank control.

Mass spectrometry conditions: A Waters Xevo G2 QTOF system (Waters) equipped with Z‐Spray ESI source and MassLynx 4.1 was used for MS data acquisition. Negative ion mode was applied to acquire data under a mass ranging 50–1200 Da with a scan time of 0.2 s and detected for 20 min, and both low‐energy (function 1, collision energy 6 V) and high‐energy (function 2, 45 V) scan functions were used. The capillary voltages were set at 2.0 kV, and the sampling cone voltage was 45 V. The source and desolvation temperatures were 100 and 350°C, respectively. The desolvation gas flow rate was 600 L/h, and the cone gas flow rate was 50 L/h.

Mass data processing and analysis: MassLynx 4.1 was used to acquire UPLC‐Q‐TOF‐MS data.

### 2.4 Experimental animals and treatment

Male C57BL/6J mice (6–8 weeks of old) were purchased from Fujian Medical University. Mice were provided with food and water ad libitum at a controlled temperature of 20 ± 2°C, with a 12‐h light/dark cycle. This study was carried out in accordance with the recommendations of the Ethical Committee for the National Laboratory Animal Act. The protocol was approved by the Animal Care and Use Committee of Fujian Medical University (2019–0129).

After 1‐week acclimation to laboratory conditions, mice were fed a HFD (60 kcal% fat; Research Diets) for 3 months to induce obesity, or normal chow diet (9% fat; Research Diets) as a control group. Mice were randomly divided into six groups (*n* = 9 per group): the chow diet group (CW CON), the chow diet + high dose of FMS treated group (CW FMSH, 100 mg kg^−1^ day^−1^), the high‐fat‐diet control group (HFD CON), the high‐fat‐diet + low dose of FMS treated group (HFD FMSL, 50 mg kg^−1^ day^−1^), the high‐fat‐diet + high dose of FMS treated group (HFD FMSH, 100 mg kg^−1^ day^−1^), and the high‐fat‐diet + Orlistat group (Orlistat, 10 mg kg^−1^ day^−1^). The control groups were intragastrically administered 0.9% normal saline only, whereas the other groups were intragastrically administered 50, 100 mg/kg FMS or Orlistat for 8 weeks. After mice were anesthetized with chloral hydrate (3 ml/kg), blood samples were obtained from the abdominal aorta and stored at −80°C after centrifugation at 2000 g for 15 min at 4°C. At the end of the experiment, all mice were fasted overnight and sacrificed via cervical dislocation. Liver tissues, WAT, and BAT were excised, washed, and weighed, frozen in liquid nitrogen, and kept at −80°C.

### 2.5 Physiological index

The body weight of every mouse was recorded daily, and the body temperature was obtained on three consecutive days. For the cold stress experiment, mice were singly caged at a cold temperature (4°C) with food and water for 6 h, and the core body temperature was recorded every hour.

### 2.6 Biochemical marker assay

The levels of alanine aminotransferase (ALT), aspartate aminotransferase (AST), triglyceride (TG), and total cholesterol (TC) in serum were examined using a Hitachi 7080 automatic biochemical analyzer (Hitachi 7080). The levels of IL‐1, IL‐6, and TNF‐α in serum were analyzed using ELISA kits according to the manufacturer's instructions (ABclonal Biotech).

### 2.7 Histological analysis

For hematoxylin and eosin (H&E) staining, liver and adipose tissues were fixed in 4% paraformaldehyde, embedded in paraffin, and cut into 7 μm sections. Sections were stained and analyzed at 100× magnification using a microscope.

### 2.8 Tolerance test

For the glucose tolerance test (GTT), mice were fasted for 16 h, then received an intraperitoneal injection of glucose (1.5 g/kg). For the insulin tolerance test (ITT), mice were fasted for 6 h, then received an intraperitoneal injection of human insulin (2 IU/kg). Blood samples were collected from the tip of the tail. Blood glucose levels were measured at 0, 15, 30, 60, 90, and 120 min after the injection of glucose or insulin, and determined by a glucose meter (ABclonal Biotech).

### 2.9 RNA isolation and quantitative RT‐PCR

Total RNA was extracted from 20 to 30 mg of mouse liver and adipose tissues using TRIpure reagent (Aidlab). The cDNA was synthesized using a HiFiScript cDNA synthesis kit (Cwbio). PCR was performed using PowerUp^™^ SYBR^™^ Green Master Mix (Thermo Fisher) on a QuantStudio^™^ 6 Flex system. The primers used in this article are listed in Table S1.

### 2.10 Statistical analysis

**FIGURE 1:**
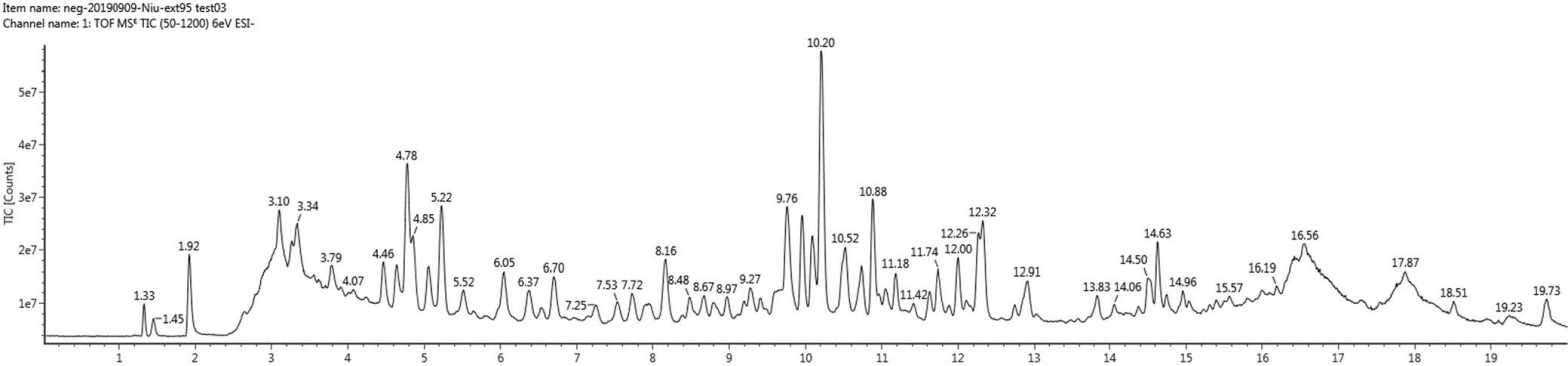
UPLC‐Q‐TOF‐MS base peak intensity chromatograms. Total ion chromatograms based on UPLC‐ESI‐Q‐TOF‐MS in the negative ion mode of FMS

All data are presented as mean ± *SD*. Statistical analysis was performed using GraphPad Prism 7.0 software. The one‐way ANOVA followed by Duncan's test and Student's *t*‐test were employed for multiple comparisons and individual comparisons. Values of *p* < .05 were considered statistically significant (Figure 1).

## 3 RESULTS

### 3.1 Identification of flavonoid compounds in FMS

**FIGURE 2:**
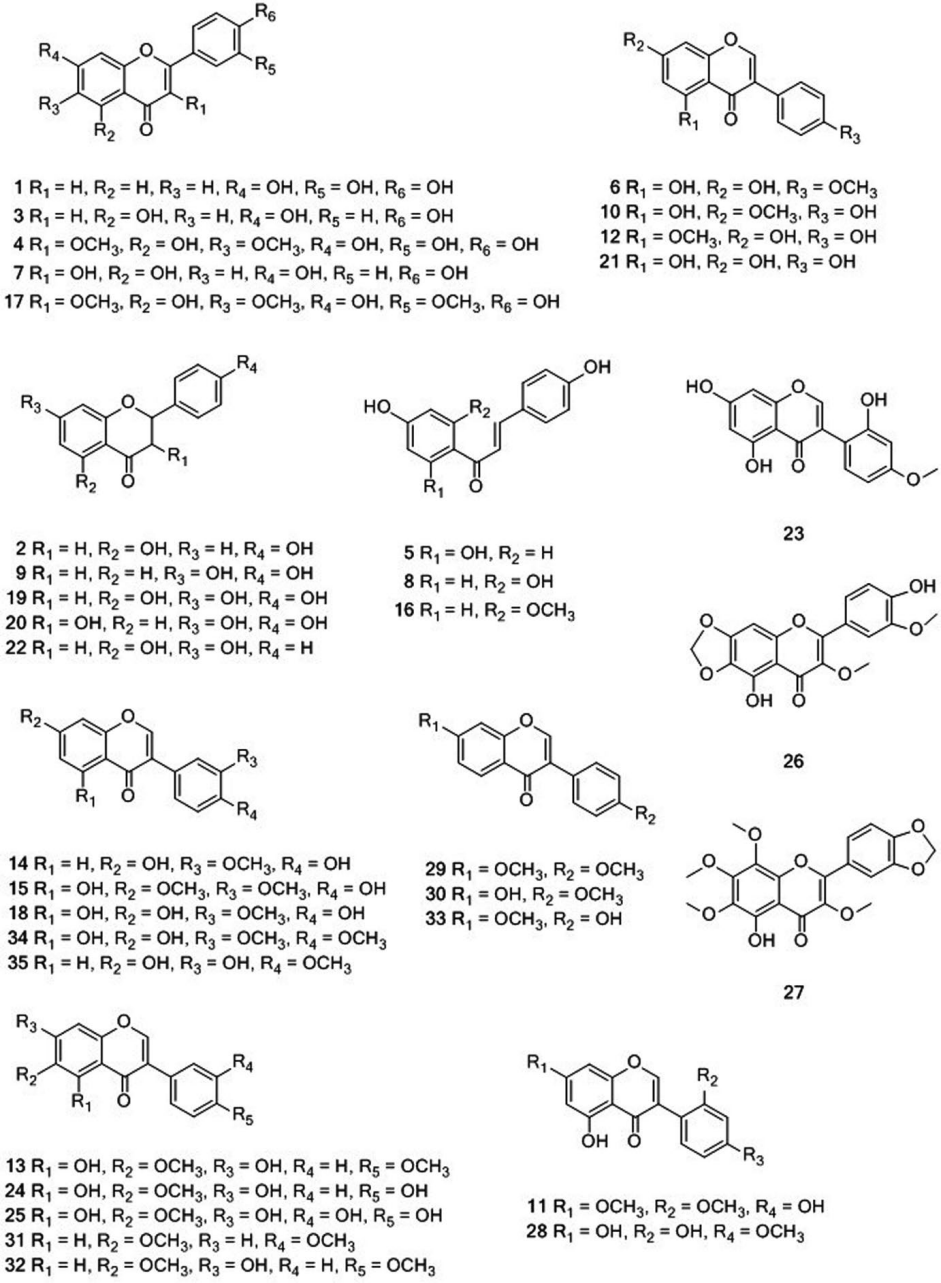
Chemical structures of the flavonoids identified from *M. speciosa* root extract

Thirty‐five peaks were identified in FMS by UPLC‐Q‐TOF‐MS, either by comparing their retention times, accurate molecular ions, and characteristic fragment ions with those of the reference compounds, or by comparing them with the reported data on the same compounds in the literature, an online TCM Chinese database [UNIFI1.7], and ChemSpider (Figure 1, Table 1). Their structures are shown in Figure 2. Among these, the fragmentation patterns of 10 flavonoids are detailed in the supplementary data. There are two general fragmentation patterns of flavonoids, and fragmentation is usually caused by the cleavage of ring C, the subsequent loss of CO, and/or CH_3_ loss from a methoxy group substituted on benzene ring A or ring B. Another fragmentation pattern due to a broken linkage between ring B and ring C was also found here.

**TABLE 1:** Mass spectrometry data and identification of *M. speciose*

Peak No.	Rt^a^ (min)	Formula	Negative ion mode of ESI‐MS (*m*/*z*)		Molecular weight		Mass error (ppm)	Identification
			Quasi‐molecular ion	MS^2^ ions	Observed	Theoretical		
1	4.64	C_15_H_10_O_5_	269.0451 [M‐H]^−^	161.0246 135.0455 133.0293	270.0526	270.0528	−1.0	7,3′,4′‐trihydroxy‐flavone
2	5.06	C_15_H_12_O_4_	255.0650 [M‐H]^−^	135.0083 117.0344	256.0720	256.0736	−4.1	5,4′‐dihydroxy‐flavone
3	5.18	C_15_H_10_O_5_	269.0452 [M‐H]^−^	161.0245 133.0291	270.0525	270.0528	−1.1	5,7,4′‐trihydroxy‐flavone
4	5.78	C_17_H_14_O_8_	345.0613 [M‐H]^−^	–	346.0685	346.0689	−1.0	Axillarin
5	6.05	C_15_H_12_O_4_	255.0651 [M‐H]^−^	135.0086	256.0731	256.0736	−0.2	5,7,4′‐trihydroxy‐chalcones
6	7.11	C_16_H_12_O_5_	283.0611 [M‐H]^−^	269.0453 149.0608 147.0452 135.0088 131.0502 109.0295	284.0684	284.0685	−0.3	5,7‐dihydroxy−4′‐methoxy‐isoflavone
7	7.44	C_15_H_10_O_6_	285.0411 [M‐H]^−^	239.0674 229.0846 167.0322 123.0404	286.0484	286.0477	2.2	Kaempferol
8	7.55^b^	C_15_H_12_O_4_	255.0666 [M‐H]^−^	135.0088 117.0346	256.0739	256.0736	1.2	Isoliquiritigenin
9	9.56^b^	C_15_H_12_O_4_	255.0650 [M‐H]^−^	135.0087 117.0345	256.0723	256.0736	−4.9	Liquiritigenin
10	7.57	C_16_H_12_O_5_	283.0625 [M‐H]^−^	269.0453 135.0086	284.0698	284.0685	4.7	5,4′‐dihydroxy−7‐methoxy‐isoflavone
11	7.77	C_16_H_12_O_7_	315.0501 [M‐H]^−^	302.0790 192.0428 123.0451	316.0570	316.0583	−2.8	Cajanol
12	8.10	C_16_H_12_O_5_	283.0615 [M‐H]^−^	269.0453 135.0086 131.0502	284.0688	284.0685	1.1	7,4′‐dihydroxy−5‐methoxy‐isoflavone
13	8.20	C_17_H_14_O_6_	313.0713 [M‐H]^−^	299.0561 181.0142 165.0193 149.0608 147.0452 139.0401 131.0502	314.0786	314.0790	−1.4	Irisolidone
14	8.70	C_16_H_12_O_5_	283.0602 [M‐H]^−^	269.0452 165.0557 147.0452 163.0401 135.0088 119.0139 93.0346	284.0674	284.0685	−3.7	5,4′‐dihydroxy−3′‐methoxy‐isoflavone
15	8.86	C_17_H_14_O_6_	313.0712 [M‐H]^−^	203.0807 197.0441 167.0337	314.0781	314.0790	−2.4	5,4′‐dihydroxy−7,3′‐dimethoxy‐isoflavone
16	8.91^b^	C_16_H_14_O_4_	269.0808 [M‐H]^−^	213.0898	270.0881	270.0892	−4.2	4′,4′‐dihydroxy−2′‐methoxychalcone
17	9.30	C_18_H_16_O_8_	359.0763 [M‐H]^−^	344.2545 223.1687 169.0244	360.0836	360.0845	−2.6	Quercetagetin 3,3′,6‐trimethyl ether
18	9.35	C_16_H_12_O_6_	299.0549 [M‐H]^−^	285.0405 165.0557 163.0401 151.0037 147.0452 135.0452 107.0502 94.0424	300.0622	300.0634	−4.0	5,7,4′‐trihydroxy−3′‐methoxy‐isoflavone
19	9.46	C_15_H_12_O_5_	271.0598 [M‐H]^−^	–	272.0671	272.0685	−5.1	Naringenin
20	9.86	C_15_H_12_O_5_	271.0604 [M‐H]^−^	–	272.0677	272.0685	−3.0	Garbanzol
21	9.93^b^	C_15_H_10_O_5_	269.0446 [M‐H]^−^	161.0246 133.0290	270.0519	270.0528	−3.5	Genistein
22	10.21	C_15_H_12_O_4_	255.0652 [M‐H]^−^	177.0193	256.0730	256.0736	−0.2	5,7‐dihydroxy‐flavanonol
23	10.30	C_16_H_12_O_6_	299.0547 [M‐H]^−^	285.0405 165.0557 163.0401 151.0037 147.0452 135.0088 109.0295	300.0620	300.0634	−4.7	2′‐hydroxy biochanin A
24	10.63	C_16_H_12_O_6_	299.0557 [M‐H]^−^	285.0404 256.0455 151.0036	300.0630	300.0634	−1.3	Tectorigenin
25	10.68	C_16_H_12_O_7_	315.0500 [M‐H]^−^	125.0240	316.0573	316.0583	−3.1	Irilin D
26	10.78	C_17_H_12_O_6_	357.0606 [M‐H]^−^	–	358.0679	358.0689	−2.7	5,4′‐dihydroxy−3,3′‐dimethoxy−6,7‐methylenedioxyflavone
27	10.92	C_20_H_18_O_9_	401.0872 [M‐H]^−^	121.0292	402.0945	402.0951	−1.4	5‐dihydroxy−3,6,7,8‐tetramethoxy−3′,4′‐methylenedioxyflavone
28	11.18	C_16_H_14_O_6_	301.0711 [M‐H]^−^	–	302.0784	302.0790	−2.2	Ferreirin
29	11.41	C_16_H_10_O_5_	281.0457 [M‐H]^−^	253.0499 251.0339 121.0290	282.0530	282.0528	0.6	7,4′‐dimethoxy‐isoflavone
30	11.72^b^	C_16_H_12_O_4_	267.0662 [M‐H]^−^	253.0505 135.0455 133.0442	268.0735	268.0736	−0.4	Formononetin
31	11.85	C_17_H_14_O_5_	297.0753 [M‐H]^−^	254.0570	298.0826	298.0841	−5.3	6‐hydroxy−7,4′‐dimethoxy‐isoflavone
32	12.11	C_17_H_14_O_5_	297.0760 [M‐H]^−^	254.0571	298.0833	298.0841	−2.9	7‐hydroxy−6,4′‐dimethoxy‐isoflavone
33	12.26	C_16_H_12_O_4_	267.0660 [M‐H]^−^	253.0506 149.0244 135.0452 133.0441 117.0346 107.0502	268.0733	268.0736	−0.3	4′‐hydroxy−7‐methoxy‐isoflavone
34	12.33	C_17_H_14_O_6_	313.0711 [M‐H]^−^	161.0446	314.0780	314.0790	−2.2	7,5‐dihydroxy−3′4′‐dimethoxy‐isoflavanone
35	14.00^b^	C_16_H_12_O_5_	283.0600 [M‐H]^−^	269.0450 165.0556 147.0451	284.0673	284.0685	−4.3	Calyclosin

^a^ The retention time was acquired from UPLC Q‐TOF MS.

^b^ Confirmed in comparison with authentic standards.

### 3.2 FMS inhibited the weight gain and improved the glucose tolerance and insulin sensitivity of obese mice

**FIGURE 3:**
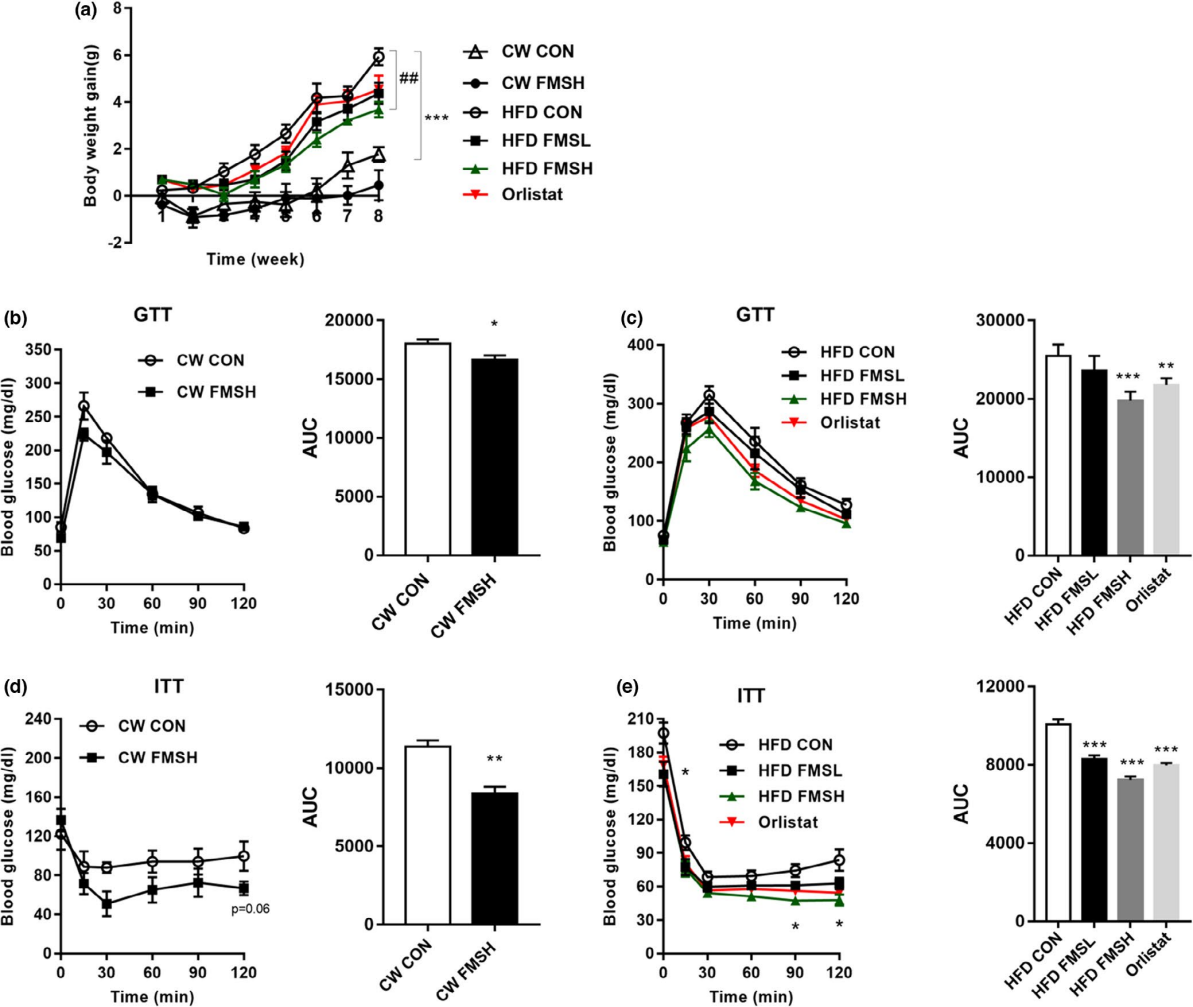
FMS inhibited the weight gain and improved glucose tolerance and insulin sensitivity of HFD‐induced obesity mice. The body weight gain of four groups mice. ***p < .001, compared with the CW CON group. ^#^ *p* < .05, compared with the HFD CON group (a). The glucose tolerance test (GTT) and the insulin tolerance test (ITT) were performed (b‐e). AUC: area under curve. HFD CON represents mice fed a high‐fat diet; HFD FMSL represents mice fed a high‐fat diet and 50 mg/kg FMS; HFD FMSH represents mice fed a high‐fat diet and 100 mg/kg FMS; CW CON represents mice fed a chow diet; CW FMSH represents mice fed a chow diet and 100 mg/kg FMS. Values are means ± *SD* (*n* = 9). *p < .05, **p < .01, and ***p < .001

To investigate the effect of FMS on obesity, an HFD‐induced obese mouse model was established. As shown in Figure 3a, HFD significantly increased the body weight gain of HFD CON group mice, compared with that of CW CON group mice (*p* < .001). At the same time, FMS and Orlistat treatment markedly inhibited the weight gain of HFD‐fed mice (Figure 3a, *p* < .01). Further, the glucose tolerance test (GTT) and the insulin tolerance test (ITT) were performed. The result shows that HFD‐fed mice had a significantly higher fasting blood glucose level after the injection of glucose, indicating a certain degree of hyperglycemia. The high dose of FMS and Orlistat treatment effectively inhibited the increase in blood glucose after the injection of glucose (Figure 3b,c). Moreover, consistent with the Orlistat group, the blood glucose content and AUC (area under the curve) for the ITT in HFD FMSL and HFD FMSH group mice were significantly lower than those in HFD CON group mice (Figure 3d,e, *p* < .001). These results indicate that FMS inhibited the weight gain and improved the glucose tolerance and insulin sensitivity of HFD‐induced obese mice.

### 3.3 FMS inhibited the weight gain of WAT and upregulated lipolysis, fatty acid oxidation, and OXPHOS pathway–related genes

**FIGURE 4:**
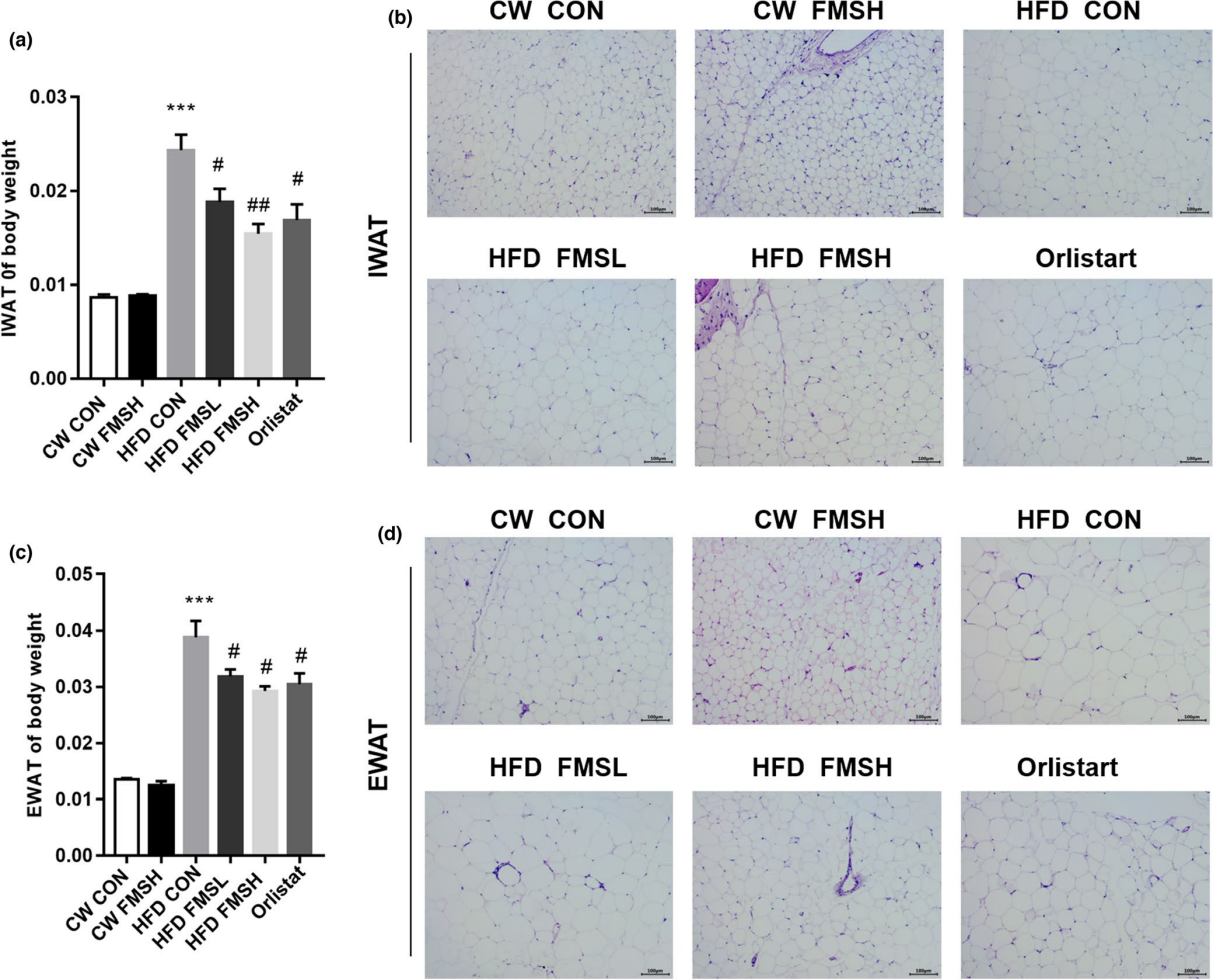
FMS inhibited the weight gain of inguinal white adipocyte tissue (IWAT) and epididymal white adipose tissue (EWAT). The effect of FMS on the weight gain of IWAT and EWAT in control and HFD group mice was analyzed (a and c). The effect of FMS on the morphological changes in IWAT and EWAT were examined through hematoxylin and eosin (H&E) stain (scale bar: 100 µm; b and d). Results are presented as mean ± *SD* (*n* = 9). ***p < .001, compared with the CW CON group; ^#^ *p* < .05, ^##^ *p* < .01, compared with the HFD CON group

Further, inguinal white adipocyte tissue (IWAT) and epididymal white adipose tissue (EWAT) were isolated and weighed. The results show that the weight of IWAT and EWAT were both markedly increased in HFD group mice, compared with CW CON group mice (Figure 4a,c); while FMS markedly inhibited the weight gain of IWAT and EWAT in HFD group mice, it has no effect on CW mice (Figure 4a,c). At the same time, histological analysis of IWAT and EWAT showed that HFD significantly promoted adipocyte hypertrophy and increased fat cell area in comparison with the CW CON group, while the administration of FMS partly prevented this phenomenon (Figure 4b,d).

**FIGURE 5:**
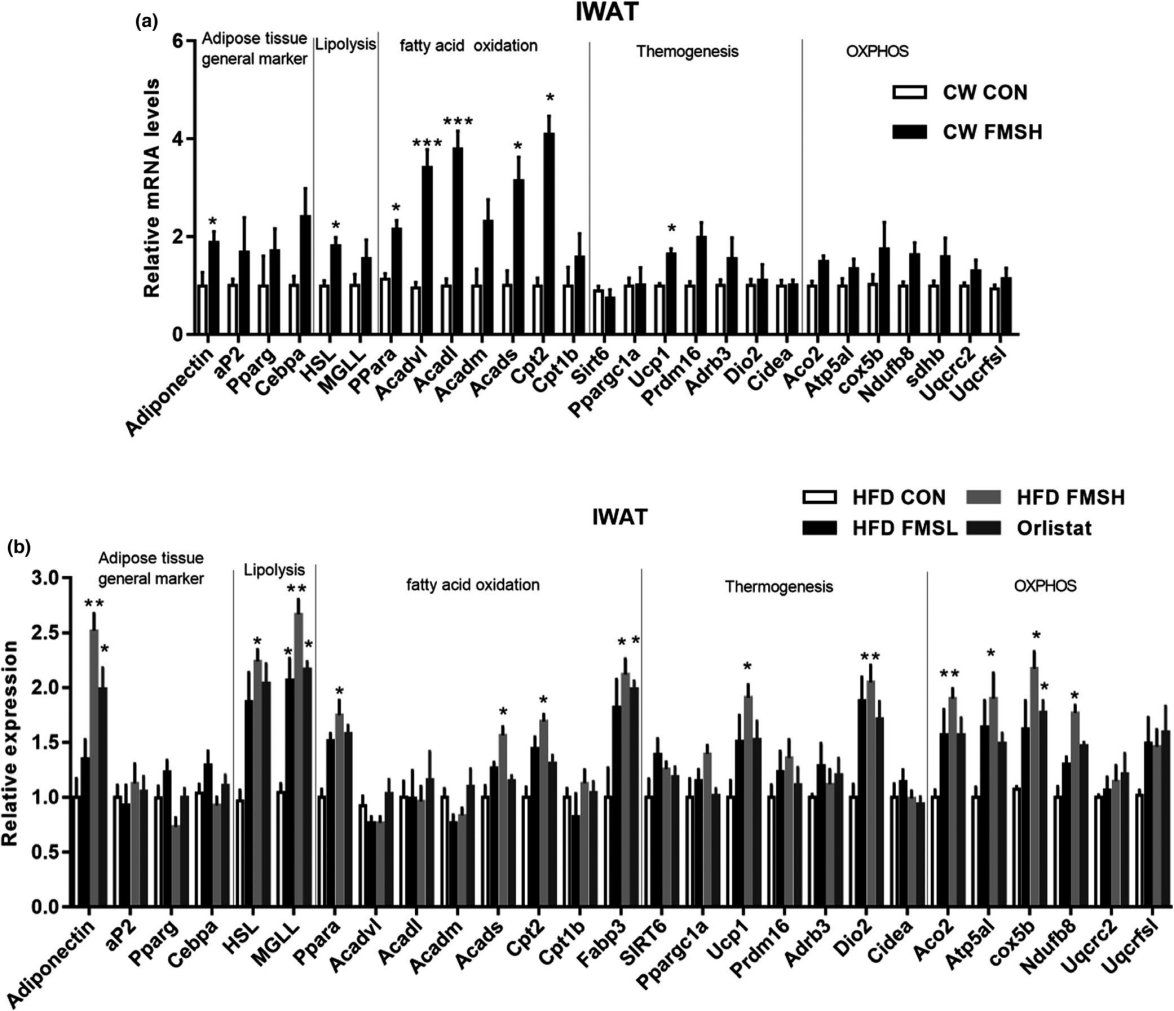
FMS upregulated lipolysis‐ and thermogenesis‐related genes in inguinal white adipocyte tissue (IWAT). The effect of FMS on the expression levels of adipose tissues general markers, lipolysis, fatty acid oxidation, thermogenesis and OXPHOS‐related genes in IWAT tissues was examined (a, compared with the CW CON group; b, compared with the HFD CON group). Results are presented as mean ± *SD* (*n* = 9). *p < .05, **p < .01, ***p < .001

Next, to investigate the underlying mechanisms of FMS on obese mice induced by HFD, the expression levels of adipose tissue markers, lipolysis, fatty acid oxidation, thermogenesis, and OXPHOS‐related genes were examined in IWAT tissues. As shown in Figure 5a,b, FMS upregulated the expression of adiponectin (an adipose tissue marker), HSL (hormone‐sensitive lipase, a lipolysis‐related gene), Cidea (cell death‐inducing DFFA‐like effector) and fatty acid oxidation–related genes PPARα (peroxisome proliferator‐activated receptor alpha), ACADS (acyl‐CoA dehydrogenase short chain), and CPT2 (carnitine palmitoyltransferase 2) in IWAT tissue of both CW FMS and HFD FMS group mice. While oxidative phosphorylation (OXPHOS) pathway–related genesAco2 (aconitase 2), Atp5al (ATP synthase F1 subunit alpha), cox5b (cytochrome c oxidase subunit 5B), and Ndufb8 (NADH: ubiquinone oxidoreductase subunit B8) were significantly upregulated in IWAT tissue in only HFD FMS group mice (Figure 5b). These results suggest that FMS mainly induced the activation of fatty acid oxidation in IWAT tissues of chow diet feeding mice, while it induced the activation of lipolysis, fatty acid oxidation, and OXPHOS pathway in IWAT tissue of ****HFD‐fed mice.

### 3.4 FMS promoted the generation of BAT and upregulated thermogenesis‐related genes in obese mice

**FIGURE 6:**
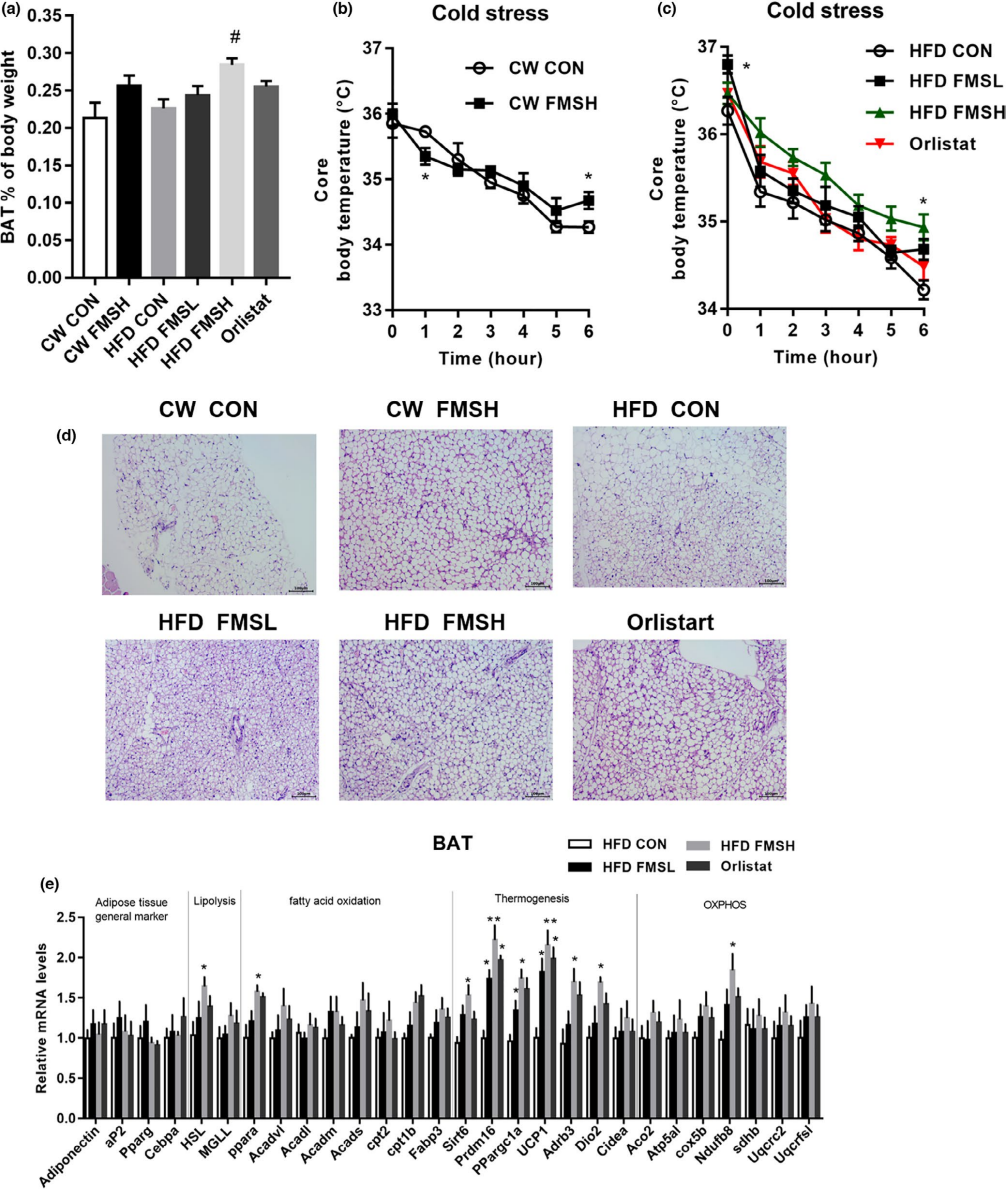
FMS promoted the generation of brown adipose tissue (BAT) and upregulated thermogenesis‐related genes. The effect of FMS on the weight gain of BAT in control and high‐fat diet (HFD) group mice was analyzed. Values are means ± *SD* (*n* = 9), ^#^ *p* < .05, compared with the HFD CON group (a). The body temperature of cold stress test was collected at seven time points (0, 1, 2, 3, 4, 5, and 6 h; b and c). The effect of FMS on the morphological changes in BAT (scale bar: 100 µm; d). The expression levels of adipose tissues general markers, lipolysis, fatty acid oxidation, thermogenesis and OXPHOS‐related genes in BAT tissues were examined in HFD group mice (e). Results are presented as mean ± *SD* (*n* = 9), *p < .05, compared with the HFD CON group

As shown in Figure 6a, FMSH markedly increased the content of BAT in HFD‐fed mice, but had no significant effect on CW mice. Orlistat had no effect on BAT content in HFD‐fed mice (Figure 6a). The cold tolerance test showed that the body temperature of HFD group mice significantly declined during 6 h of exposure to 4°C, while FMS partly inhibited the drop of body temperature in HFD FMSL and HFD FMSH group mice, compared with HFD CON group mice (Figure 6b,c). This result reveals that FMS may facilitate adaptation to cold exposure. Histological analysis of BAT revealed that FMS significantly improved adipocyte hypertrophy and fat cell area of HFD group mice (Figure 6d). In addition, thermogenesis‐related genes Sirt6 (sirtuin 6), Prdm16 (PR domain containing 16), Ppargc1A (peroxisome proliferative‐activated receptor, gamma, coactivator 1 alpha), and UCP1 (uncoupling protein‐1) were upregulated in BAT tissue of HFD FMS group mice, compared with HFD CON group mice (Figure 6e). Interestingly, FMS induced only thermogenesis‐related Cidea gene overexpression in the BAT of mice fed a chow diet (Figure S1a). It is speculated that FMS promoted the production of BAT and the thermogenesis program in the BAT of obese mice.

### 3.5 FMS ameliorated HFD‐induced abnormal liver function and inflammation

**FIGURE 7:**
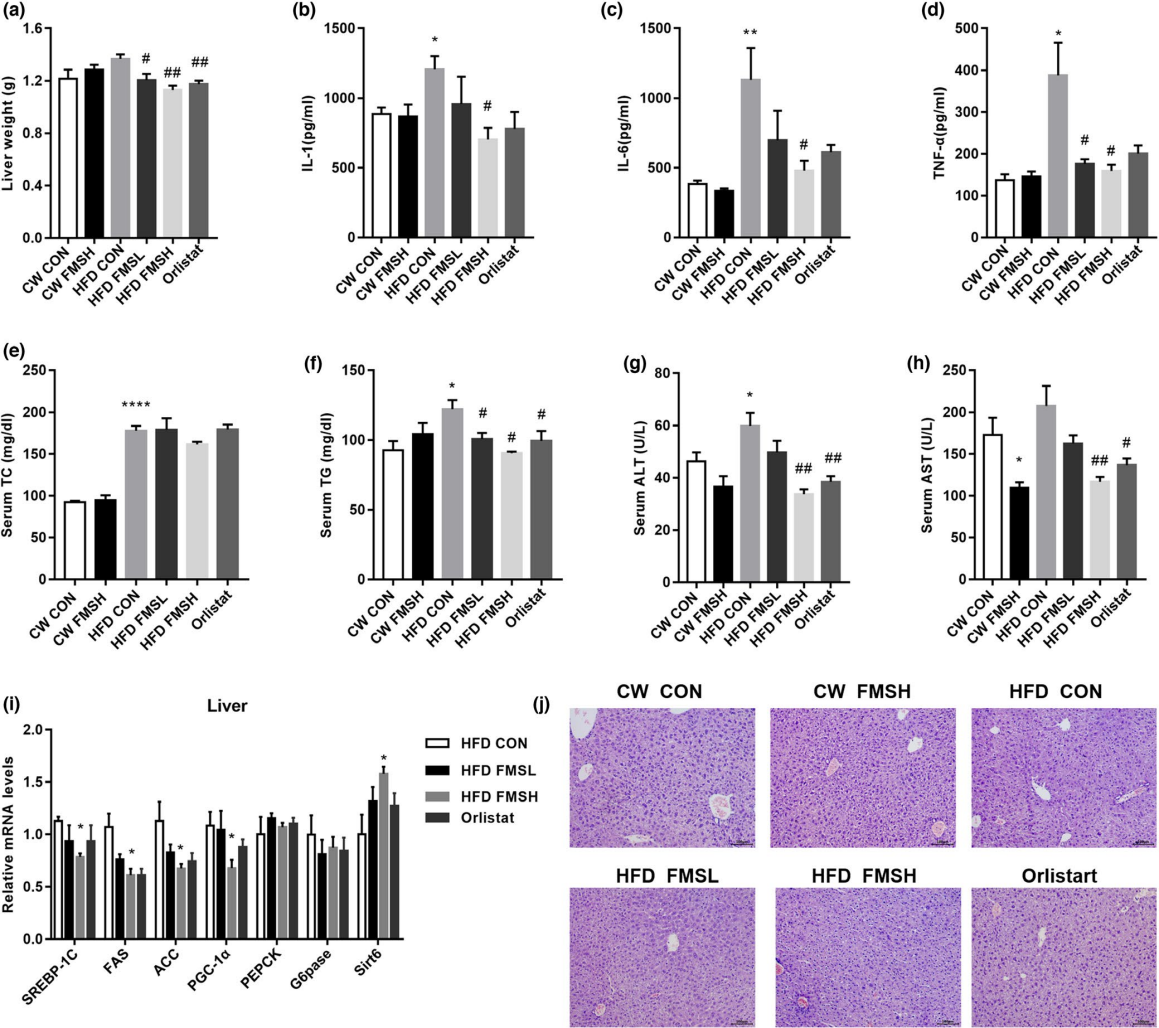
FMS ameliorated high‐fat diet (HFD)–induced abnormal liver function and inflammation. The relative weight of liver in CW and HFD groups mice were analyzed (a, ^#^ *p* < .05, ^##^ *p* < .01, compared with the HFD CON group). The levels of pro‐inflammatory cytokines IL‐1, IL‐6, and TNF‐α in serum were examined using ELISA kits (b‒d, *p < .05, **p < .01, compared with the CW CON group; ^#^ *p* < .05, compared with the HFD CON group). The levels of TC, TG, ALT, and AST in serum were examined through ELISA kits (e‒h, *p < .05, ****p < .0001, compared with the CW CON group; ^#^ *p* < .05, ^##^ *p* < .01 compared with the HFD CON group). The mRNA levels of sterol regulatory element binding protein 1c (SREBP‐1C), fatty acid synthase (FAS), acetyl‐CoA carboxylase (ACC), peroxisome proliferator‐activated receptor‐gamma coactivator‐1 alpha (PGC‐1α), phosphoenolpyruvate carboxykinase (PEPCK), glucose‐6‐phosphatase (G6pase), and sirtuin 6 (Sirt6) in liver tissue were tested using real‐time PCR (i, *p < .01, compared with the HFD CON group). The effect of FMS on liver tissue were analyzed by H&E staining, scale bar: 100 µm (j). Results are presented as mean ± *SD* (*n* = 9)

Epidemiological data showed that obesity is a major risk factor for fatty liver disease. We found that FMS and Orlistat both obviously suppressed the weight of liver tissue in obese mice, compared with HFD CON group mice (Figure 7a). Moreover, ELISA results reveal that FMSH effectively inhibited HFD‐induced upregulation of inflammatory‐related adipokines IL‐6 and TNF‐α (Figure 7b‐d). In addition, the levels of TC, TG, ALT, and AST were examined in the serum samples. Among them, ALT and AST are sensitive markers of liver damage. The results show that HFD increased the levels of TC, TG, ALT, and AST in HFD CON group mice compared with the CW CON group (Figure 7e‐h), while FMSH significantly inhibited the increase in TG, ALT, and AST (Figure 7e‐h). Moreover, FMSH dramatically inhibited the expression of SREBP‐1C (sterol regulatory element‐binding transcription factor 1), FAS (fatty acid synthase), and ACC (acetyl‐CoA carboxylase) in liver tissues and markedly increased the expression of Sirt6 in liver tissues (Figure 7i). However, it did not affect the expression of these genes in CW feeding mice (Figure S1b). Further, H&E staining revealed that treatment with FMS significantly alleviated HFD‐induced macrovesicular steatosis in liver tissues (Figure 7j). Hence, it is suggested that FMS alleviated HFD‐induced abnormal liver function and inflammation.

## 4 DISCUSSION

In the present study, we found that both the high and low doses of FMS inhibited body weight gain, the content of WAT, and improved the glucose tolerance and insulin sensitivity of HFD‐induced obese mice. Obesity is strongly associated with the development of insulin resistance, which in turn plays a crucial role in the pathogenesis of obesity‐associated complications, including type 2 diabetes, metabolic syndrome components, and cardiovascular diseases (Barazzoni et al., 2018). Adipose tissue, skeletal muscle, and liver are insulin‐sensitive tissues, and excessive lipid accumulation leads to local inflammation and insulin resistance (Wiklund et al., 2016).

White adipose tissue has a crucial role in regulating systemic energy homeostasis, it is responsible for the breakdown of stored fat into fatty acids; subsequently, fatty acids are broken down to generate acetyl‐CoA in mitochondria and peroxisomes, while acetyl‐CoA is completely oxidized into CO_2_ and H_2_O, thereby releasing large quantities of energy (Han van der Kolk, et al., 2017). This multistep process goes through several stages, including lipolysis, fatty acid oxidation, and OXPHOS. In our study, FMS induced the upregulation of HSL (a lipolysis‐related gene), fatty acid oxidation–related genes (PPara, Acadvl, Acadl, Acads, and Cpt2), and OXPHOS‐related genes (Aco2, Atp5al, and Ndufb8) in IWAT tissues of HFD FMS group mice. These results verify that FMS promotes the energy metabolism of WAT in obese mice.

Numerous studies have revealed that promoting the formation of BAT could increase energy expenditure and inhibit weight gain in obese patients (Piao et al., 2018; Zhang et al., 2013). In this study, we found that a high dose of FMS promoted the relative weight of BAT in HFD‐induced obese mice, while Orlistat has no significant effect on the BAT content. BAT mediates adaptive thermogenesis depending on UCP1, an uncoupling protein located in the inner mitochondrial membrane (Ikeda et al., 2017). UCP1 uncouples the respiratory chain, allowing for fast substrate oxidation with a low rate of ATP production, thereby increasing energy expenditure (Alvarez‐Crespo et al., 2016). It has been reported that enforced expression of UCP1 could relieve obesity in obese animal models, while knockdown of UCP1 in mice results in obesity (Feldmann et al., 2009). In the present study, we found that the administration of FMS increased the production of BAT and the expression of UCP1. Moreover, we found that FMS increased the adaptation of obese mice to cold exposure. Thermogenesis‐related genes (Sirt6, prdm16, and PPargc1A) were upregulated by FMS in HFD‐induced obese mice. Prdm16 is a transcription coregulator that regulates the development of brown adipocytes (Trajkovski et al., 2012). It can activate brown adipocytes to produce heat, and play an important role in thermogenic processes through upregulating thermogenic genes such as UCP1 (Iida et al., 2015). The overexpression of Prdm16 could protect against HFD‐induced weight gain (Seale et al., 2011). PPargc1A (peroxisome proliferator–activated receptor gamma coactivator 1‐alpha) is a transcriptional coactivator that controls the genes involved in energy metabolism (Zhang et al., 2020), and it contributes to the activation of thermogenic genes that promote the browning of white adipose tissue (Roberts et al., 2014). It is strongly induced by cold exposure and induces adaptive thermogenesis (Liang & Ward, 2006). Qu et al. (2019) reported that kiwifruit seed oil has an anti‐obesity effect in HFD‐induced obese mice, as it induced adipocyte browning through the upregulation of UCP‐1, PPargc1A, and Prdm16, which is in accordance with our results. Hence, our study reveals that FMS stimulates thermogenic processes in brown adipocytes and the browning of WAT in obese mice.

Obesity is accompanied by an increase in adipose tissue and ectopic lipid deposition in the liver that causes corresponding metabolism dysfunction (Goossens & Blaak, 2015). Meanwhile, the production of inflammatory cytokines and reactive oxygen species within adipose tissue impairs hepatic function (Saltiel & Olefsky, 2017). The levels of TG, ALT, and AST in the serum were obviously downregulated by FMS administration; these are sensitive markers reflecting damage to the liver (Yang et al., 2020). In addition, FMS reduced the levels of inflammatory‐related adipokines IL‐6 and TNF‐α in the serum of HFD‐induced obese mice. IL‐6 has pro‐inflammatory activity and can increase the level of TNF‐α. TNF‐α secreted by adipose tissue is correlated with insulin resistance and the degree of obesity (Tzanavari et al., 2010). In addition, FMS administration alleviated HFD‐induced liver tissue pathological change and upregulated the expression of lipid metabolism–related genes (SREBP‐1c, FAS, and ACC) (Kim et al., 2020). SREBP‐1c is a critical transcription factor involved in regulating the synthesis of lipid in the liver. FAS, a downstream target of SREBP‐1c, is involved in fat accumulation and insulin resistance (Yang et al., 2021). The cleavage and nuclear translocation of SREBP‐1c led to a reduction in lipogenesis and lipid accumulation (Li et al., 2011). SREBP‐1c is usually upregulated in HFD‐induced obese mice (Yang et al., 2021); the inhibition of SREBP activity could attenuate hepatic steatosis and atherosclerosis in diet‐induced insulin‐resistant mice (Ho et al., 2019; Li et al., 2011). These results indicate that FMS can alleviate HFD‐induced abnormal liver function through suppressing the inflammatory response and ectopic lipid deposition in the liver.

## 5 CONCLUSION

In summary, we identified 35 flavonoids from the extract of *M*. *speciosa* root using the UPLC‐Q‐TOF‐MS analysis. Meanwhile, our study found that FMS administration reduced body weight gain, liver weight gain, IWAT, and blood glucose in HFD‐induced obese mice. The results of serum analysis show that FMS decreased the levels of TG, ALT, and AST, and the inflammatory‐related adipokines IL‐6 and TNF‐α in HFD induced obese mice. Histopathological studies of WAT, BAT, and liver tissue revealed that FMS inhibited lipid accumulation in WAT, promoted the generation of BAT, and ameliorated HFD‐induced liver dysfunction. Furthermore, the analysis of the mRNA expression of lipid metabolism–related genes suggested that FMS promoted the thermogenesis program in BAT by upregulating Sirt6, prdm16, PPargc1A, and UCP1. FMS also induced the activation of lipolysis (HSL), fatty acid oxidation (PPara, Acadvl, Acadl, Acads, and Cpt2), and OXPHOS (Aco2, Atp5al, and Ndufb8)‐related genes in IWAT tissues. These findings establish an important role for FMS in stimulating adipose tissue thermogenesis and modulating lipid metabolism. Thus, we have identified a flavonoid‐enriched extract from *M. speciosa* Champ root that may be a new potential drug or food additive for treating patients with obesity.

## CONFLICT OF INTEREST

The authors declare there are no conflict of interest for this manuscript.

## ETHICS APPROVAL

The study was conducted according to the guidelines of the National Institutes of Health Guide and approved by the Animal Care and Use Committee of Fujian Medical University (2019–0129).

## Supporting information

Supplementary Material

## Data Availability

The data underlying this article will be shared on reasonable request to the corresponding author.
