# Supplementary material for: Flavonoid‐enriched extract from Millettia speciosa Champ prevents obesity by regulating thermogenesis and lipid metabolism in high‐fat diet–induced obese C57BL/6 mice
Source: Food Sci Nutr. 2021 Nov 27;10(2):445–59. doi: 10.1002/fsn3.2664 (PMC8825741; doi:10.1002/fsn3.2664)
Supplement: Supplementary file 1 — Supplementary Material [file FSN3-10-445-s001.docx]

**SUPPLEMENTARY MATERIALS**

**SUPPORTING INFORMATION**

Additional supporting information may be found online in the Supporting information section.

**Supporting table**

Table S1 Primers used for quantitative real-time PCR

| **Primer name** | **Forward** | **Reverse** | |
| --- | --- | --- | --- |
| Adiponectin | GCCAAACACCGATTGGGGT | GGCTCCAAATCTCCTTGGTAGTT |  |
| AP2 | ATGTGCTACGGAAACTACTTTGG | CTGTAGGCATAGCGAGAGCC |  |
| PPARG | CTTGGCTGCGCTTACGAAGA | GAAAGCTCGTCCACGTCAGAC |  |
| CEBPA | GCGGGAACGCAACAACATC | GTCACTGGTCAACTCCAGCAC |  |
| HSL | AGTGCTGAGGGAATGTGACAA | TCATCTTGATAATCGTCGCAGG |  |
| MGLL | AGGCGAACTCCACAGAATGTT | ACAAAAGAGGTACTGTCCGTCT |  |
| PPARA | CCCAAGGGAGGAATAGCTTCT | CTCTGCGATGCGGTTCCAA |  |
| ACADVL | ACTACTGTGCTTCAGGGACAA | GCAAAGGACTTCGATTCTGCC |  |
| ACADL | TTTCCTCGGAGCATGACATTTT | GCCAGCTTTTTCCCAGACCT |  |
| ACADM | AACACAACACTCGAAAGCGG | TTCTGCTGTTCCGTCAACTCA |  |
| ACADS | GACTGGCGACGGTTACACA | GGCAAAGTCACGGCATGTC |  |
| CPT2 | CAGCACAGCATCGTACCCA | TCCCAATGCCGTTCTCAAAAT |  |
| CPT1B | GACTTCCGGCTTAGTCGGG | GAATAAGGCGTTTCTTCCAGGA |  |
| FABP3 | ACCTGGAAGCTAGTGGACAG | TGATGGTAGTAGGCTTGGTCAT |  |
| PPARGC1A | TATGGAGTGACATAGAGTGTGCT | GTCGCTACACCACTTCAATCC |  |
| UCP1 | GTGAACCCGACAACTTCCGAA | TGCCAGGCAAGCTGAAACTC |  |
| PRDM16 | CCACCAGCGAGGACTTCAC | GGAGGACTCTCGTAGCTCGAA |  |
| ADRB3 | TCTCTGGCTTTGTGGTCGGA | GTTGGTTATGGTCTGTAGTCTCG |  |
| DIO2 | ATGGGACTCCTCAGCGTAGAC | ACTCTCCGCGAGTGGACTT |  |
| CIDEA | TGACATTCATGGGATTGCAGAC | CATGGTTTGAAACTCGAAAAGGG |  |
| ACO2 | ATCGAGCGGGGAAAGACATAC | TGATGGTACAGCCACCTTAGG |  |
| ATP5A1 | TCTCCATGCCTCTAACACTCG | CCAGGTCAACAGACGTGTCAG |  |
| COX5B | GGAAGACCCTAATCTAGTCCCG | GTTGGGGCATCGCTGACTC |  |
| NDUFB8 | TGTTGCCGGGGTCATATCCTA | AGCATCGGGTAGTCGCCATA |  |
| SDHB | ATTTACCGATGGGACCCAGAC | GTCCGCACTTATTCAGATCCAC |  |
| UQCRC2 | AAAGTTGCCCCGAAGGTTAAA | GAGCATAGTTTTCCAGAGAAGCA |  |
| UQCRFS1 | GAGCCACCTGTTCTGGATGTG | GCACGACGATAGTCAGAGAAGTC |  |
| SIRT6 | CTCCAGCGTGGTTTTCCACA | GCCCATGCGTTCTAGCTGA |  |
| SREBP-1C | ACTTTTCCTTAACGTGGGCCT | \| TGAGCTGGAGCATGTCTTCG \| \| --- \| |  |
| FAS | GCGGGTTCGTGAAACTGATAA | GCAAAATGGGCCTCCTTGATA |  |
| ACC | GCCGTGGGGAAGGAAAAGT | CTCCTGGTTGATGCTCGACA |  |
| PGC-1α | GTTGCCTGCATGAGTGTGTG | CCCTTGGGGTCATTTGGTGA |  |
| PEPCK | GCAGTGAGGAAGTTCGTGGA | GTGAGAGCCAGCCAACAGT |  |
| G6PASE | TGAGACCGGACCAGGAAGTC | AGAATCCAAGCGCGAAACCA |  |

**Supporting data**

1. **The fragmentation pathway of 10 flavonoids compounds that identified by** **UPLC-Q-TOF-MS**

**Supporting figure**


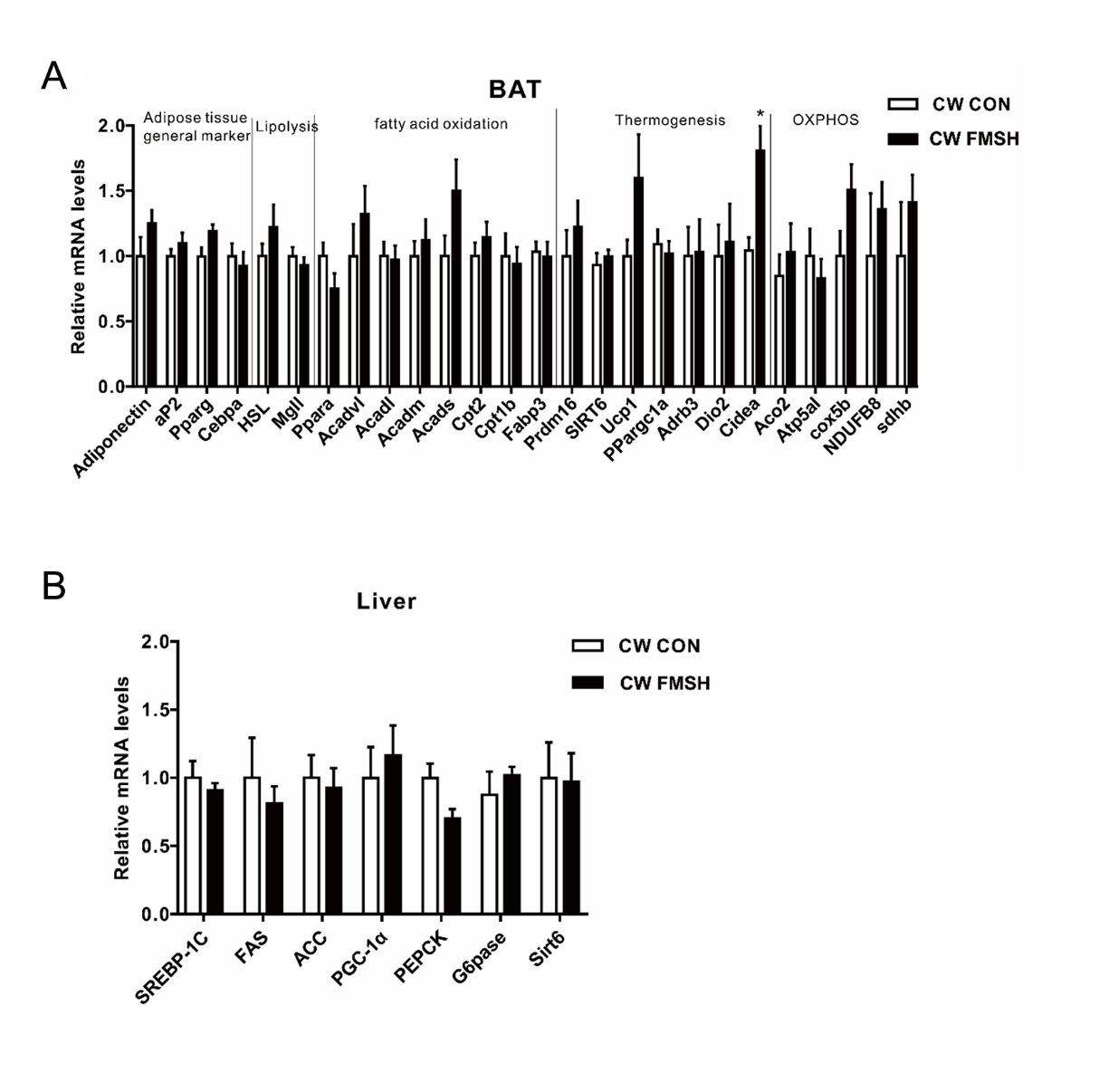


**FIGURE S1 the effect of FMSH on CW group mice.**

(A) The expression levels of adipose tissues general markers, lipolysis, fatty acid oxidation, thermogenesis and OXPHOS related genes in BAT tissues of control groups. *P <0.05, compared with CW CON group

(B) The mRNA levels of SREBP-1C, FAS, ACC, PGC-1α, PEPCK, G6pase and Sirt6 in liver tissue of CW control group and CW FMSH group were tested using real-time PCR.
